# Investigating Functional Impairment in Chronic Low Back Pain: A Qualitative Study from the Patients and Specialists’ Perspectives

**DOI:** 10.3390/jpm13061012

**Published:** 2023-06-19

**Authors:** Arturo Cuomo, Franco Marinangeli, Alberto Magni, Emiliano Petrucci, Alessandro Vittori, Marco Cascella

**Affiliations:** 1 Division of Anesthesia and Pain Medicine, Istituto Nazionale Tumori, IRCCS, “Fondazione G. Pascale”, 80131 Naples, Italy; 2 Department of Anesthesiology, Intensive Care and Pain Treatment, University of L’Aquila, Piazzale Salvatore Tommasi 1, 67100 Coppito, Italy; 3 Italian College of General Practitioners and Primary Care (SIMG), Via del Sansovino 179, 50142 Firenze, Italy; 4 Department of Anesthesia and Intensive Care Unit, San Salvatore Academic Hospital of L’Aquila, 67100 L’Aquila, Italy; 5 Department of Anesthesia and Critical Care, ARCO ROMA, Ospedale Pediatrico Bambino Gesù IRCCS, Piazza S. Onofrio 4, 00165 Rome, Italy

**Keywords:** chronic low back pain, functionality, musculoskeletal disorder, disability, chronic pain, quality of life, social cost, opioid, pain, pain therapy

## Abstract

Prompt and appropriate treatment of chronic low back pain (cLBP) is of the utmost importance for preventing relevant disability, high burden of disease, and increasing costs for the healthcare system. Recently, the concept of functional impairment has been associated with any type of chronic pain, and mounting attention has been paid to extending the aims of treatment beyond mere pain remission, including restoration of working capacity, everyday functioning, mobility, and quality of life. Nevertheless, a shared definition of functionality is still lacking. Notably, different specialists involved in the treatment of cLBP such as general practitioners, orthopedists, pain therapists, and physiatrists, and patients themselves have different opinions on what “functional impairment” actually means. On these premises, a qualitative interview study was performed to investigate how the concept of “functionality” is interpreted by different specialists involved in the management of cLBP, and by patients. Overall, all different specialists agreed that functionality should be assessed in clinical practice. However, in spite of several instruments available for evaluating functionality, no homogeneity of behavior is observable.

## 1. Introduction

Chronic low back pain (cLBP) is a common and persistent health problem that affects a significant portion of the population worldwide [1]. The condition can cause considerable disability, resulting in a heavy burden on healthcare systems and increased costs [2]. Thus, it is imperative to prioritize the timely and appropriate treatment of cLBP to ensure better outcomes, reduced healthcare costs [3], and improved health-related quality of life (HR-QoL) for patients [4].

Recently, there has been an increasing focus on expanding the goals of cLBP treatment beyond just alleviating pain. This includes improving working capacity, daily functionality, mobility, and overall HR-QoL [5,6,7,8,9,10,11].

In particular, the International Classification of Diseases (ICD)-11 underlined the concept of functional impairment associated with any type of chronic pain, including cLBP [12]. Indeed, in the ICD-11, pain severity is graded not only on the basis of intensity and distress but also on functional impairment [12]. However, a shared definition of functionality is still lacking, and different specialists involved in the treatment of cLBP including general practitioners (GPs), orthopedists, pain therapists, and physiatrists, as well as patients themselves may have different opinions on what “functional impairment” actually means. This is one among the multiple gaps observed in the management of chronic pain [13,14]. 

Several strategies can be used to study this problem and provide answers that can be useful for improving outcomes. For this aim, qualitative research can be a valid opportunity [15]. Well-designed qualitative research has been advocated in order to investigate complex and multifaceted issues in the management of chronic cancer and no-cancer pain [16,17]. This approach is based upon in-depth interviews, conducted by qualified specialists from the sociology/psychology field with a well-identified and characterized sample. The latter can include either healthcare providers, patients, or both. [18].

On these bases, we conducted qualitative research to investigate how the concept of “functionality” is interpreted by different specialists involved in the management of cLBP, and by patients themselves.

## 2. Materials and Methods

The study comprised two different surveys; the former was directed to physicians handling chronic low back pain (cLBP) in their practice, and the latter involved patients with cLBP. The investigation was performed in compliance with the EphMRA code of conduct. Every participant, including clinicians and patients, provided voluntary and informed consent for data collection and usage, based on a comprehensive comprehension of the study’s objectives and data collection purposes.

### 2.1. Physicians’ Survey

Physicians with a broad experience in cLBP management who work in different Italian geographical areas of Italy were involved. These clinicians work in the community and in different settings, including general hospitals, specialized hospitals, and research hospitals, either from the national health system or the private healthcare network. Therefore, they included GPs, orthopedists, pain therapists, and physiatrists. 

Each participant was asked to write two short papers, one on chronic pain issues in cLBP patients and the other on functional recovery of cLBP. Each participant was then interviewed by an expert in qualitative research in a face-to-face meeting, with the aim to clarify opinions and issues mentioned in the papers and to explore how functionality recovery is addressed in clinical practice. Interviews were personalized based on the content of the short papers and lasted approximately 1 h.

### 2.2. Patients’ Survey

The second survey was directed to patients with cLBP. Enrolled patients belonged to different age groups (45–60, 60–74, ≥75 years) and were stratified according to their level of instruction, duration of disease (<5, 5–10, >10 years), disease severity, and specialist they are followed by.

The eligibility criteria for participation were the following:

(a) Diagnosis of cLBP. The pool of eligible patients was provided by the clinicians who participated in the survey. Clinical data were independently evaluated by two expert researchers (A.C. and F.M.).
(b) Able to understand the information describing the research study.
(c) Signed informed consent prior to filling in the survey.

The exclusion criteria fort participation were the following:

(a) Uncertain diagnosis of pain.
(b) Any cognitive impairment.
(c) Refusal to participate in research.

Each enrolled patient was interviewed by an expert of qualitative research in a face-to-face meeting lasting about 75 min, with the aim of narrating their “patient journey” with cLBP. The interview focused on the difficulties encountered along the journey.

### 2.3. Data Analysis

All texts and transcripts of interviews were analyzed using the T-Lab software (T-LAB, Roccasecca-Frosinone, Italy) to extract different word patterns or subject patterns; more frequently used words were extracted, and relationships of words related to function recovery were explored. In the first phase of the text analysis, words were converted into a standardized pattern (i.e., the infinitive form of verbs, the single male form of adjectives, etc.). The second phase of the analysis provided the frequencies of words in the texts, connections among words (which words were most often used in connection with words of interest for the study), and narrative semantic cluster analysis. 

### 2.4. Ethics Approval and Informed Consent

For this survey, approval by Ethics Committee was not required. According to the current Italian legislation for noninterventional or observational studies (Ministerial Circular No. 6, 2 September 2002), it is necessary to submit a survey for approval by an Ethics Committee only if “the study focused on problems or pathologies within which medicinal products are prescribed in the usual way in accordance with the conditions set out in the marketing authorization. The inclusion of the patient in a specific therapeutic strategy is not decided in advance by the trial protocol but is part of normal clinical practice and the decision to prescribe the medicine is completely independent of that of including the patient in the study” [19,20].

## 3. Results

### 3.1. Physicians Survey

The first survey enrolled 52 participants: 16 general practitioners (GPs) (aged 45–50, n = 4; 50–58, n = 7; 59–63, n = 5) and 36 specialists (12 orthopedists, 12 pain therapists, and 12 physiatrists; 18 of them working in general hospitals and 18 working in research hospitals). All physicians were working in different Italian areas (North Italy n = 12; Central Italy n = 19; South Italy n = 21). 

A total of 10–15% of subjects with chronic low back pain (cLBP) managed by GPs and orthopedists had moderate to severe pain. The proportion was higher for patients treated by pain therapists (40–50%) and highest for those attended by physiatrists (70–80%). 

Word analysis was carried out on 1458 words, occurring at least once, from the texts about chronic pain or functionality written by the participating physicians (Table 1). Overall, treatment (drugs) and management (“usage”, “dose”) were the main subjects in texts about cLBP. Assessment and treatment of pain were the main interest of pain therapists; orthopedists were often concerned with the causes of pain, and GPs were concerned with drug choice. Terms related to functional recovery did not spontaneously emerge. Texts about functionality were mainly concerned with the patient’s evaluation, and with the identification of the physical or social characteristics of disability, also using dedicated scales (Table 1).

Sentences used in texts about functional recovery were dealing with five main areas: (1) definition; (2) treatments; (3) aims (autonomy recover, everyday functions, physical and psychological wellbeing); (4) physical dimension (features of disability and treatment opportunities); and (5) assessment (scales and scores). 

**Figure 1:**
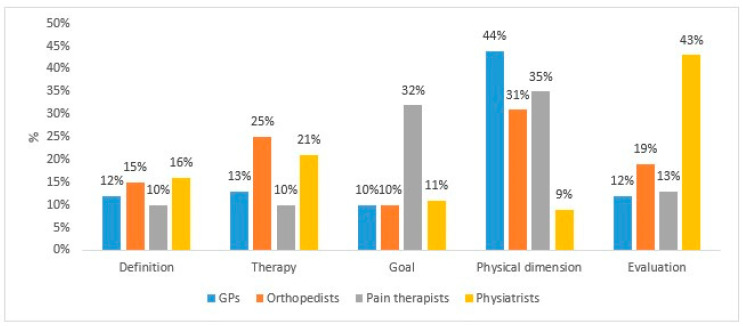
Frequency of semantic clusters as reported by different specialties.

The frequency of semantic clusters was different for participants pertaining to different specialties (Figure 1). 

The definition of function recovery was a marginal topic for participants of all specialties (10–16% of texts) (Table 2). Orthopedists, followed by physiatrists, were more often interested in treatments compared with other specialists. Functional recovery, in conjunction with an improvement of HR-QoL, was often mentioned by pain therapists as the objective of their interventions. Their aim was to enable the patient to be autonomous, to go back to her/his everyday activities, and to obtain to complete social and psychological wellbeing. The physical dimension of functioning was rarely mentioned by physiatrists but was considered very relevant by GMPs, pain therapists, and orthopedists. These physicians wrote about their commitment to finding out the site of the problem, in assessing physical limitations and movement impairment. Physiatrists were often interested in measurement tools, such as scores and scales for the evaluation of pain and the assessment of functionality. 

A deeper focus on each specialty is provided below, in a narrative way.

#### 3.1.1. General Medical Practitioners

This group of physicians used words related to many different themes. They wrote about pain, the psychological impact of cLBP, physical impairment, and psychosocial impairment. The impairment of functionality is described as a reduced ability to attend to everyday activities. It is caused by pain and consists of physical disorders preventing motility and consequently normal activity. In this perspective, function recovery means physical recovery. 

Control of pain was considered a preliminary aim, necessary to pursue a functional recovery. GPs are concerned with the choice of the best drug for pain control and feel confident in the effectiveness of the pharmacological treatment of cLBP, but they do not use drugs for direct action on functionality. They leave the patient to manage functionality by her/himself, and they generally do not refer patients to other specialists for guided physical exercise. 

#### 3.1.2. Orthopedists

Therapies and the physical dimension of disability were the most frequent subjects mentioned by orthopedists in texts about function recovery in patients with cLBP. Indeed, functional recovery was interpreted as secondary, following pain control. It consisted of recovery of mobility, and the ability to attend everyday activities with as little pain as possible. Therapy was the tool they described as useful to obtain function improvement. Therefore, in their opinion, pain control was the first necessary step toward functional recovery. Therapies mentioned in texts were mainly NSAIDs and myorelaxants.

#### 3.1.3. Pain Therapists

Texts about function recovery written by pain therapists were mainly interested in the physical dimension of health and in quality of life. The aim of the interventions was to enable the patient to work and recover her/his social dimension. In the opinion of these specialists, functional recovery is related to autonomy, social life, and psychological and physical wellbeing. 

According to most pain therapists, functional recovery corresponds to “mobility” and “everyday activities”, while according to a minority of them, it corresponds to the HR-QoL. It is mainly intended as mobility in the absence of pain; thus, the absence of pain means functional recovery.

The analgesic pharmacologic treatment is considered a useful tool for functional recovery. Some pain therapists stated that drugs are part of a complex strategy and must be used in association with physical activity, under the supervision of a physiatrist; in their opinion, drugs would not directly induce a functional recovery but enable the patient to follow a rehabilitation plan.

#### 3.1.4. Physiatrists

The main interest of physiatrists was the measurement of function. Recovery can be pursued only by starting with an attentive and objective evaluation of the patient, using assessment scales and scores for function and pain. The use of validated scales is considered mandatory. Function recovery means physical autonomy, and physical autonomy is identified as a satisfactory quality of life, according to this group of participants.

The term “functional recovery” was interpreted as recovered muscular strength and joint flexibility. These texts suggested that a patient with good motility may face everyday activity and possibly sports. The main therapeutical strategies are physical exercise and drugs, which cannot be used independently and act on two sides. In particular, drugs act on pain and physically guided activity works on functionality.

### 3.2. Patients Survey

Twenty-four patients (12 men), all living in large city areas (Milan, Rome, Naples) answered the second survey. Time from diagnosis was 1–5 years for 12 subjects, 5–10 years for six patients, and >10 years for six subjects. Nine patients were 45–60 years old, nine were 61–74 years old, and nine were ≥75 years. Although the patient’s journey may have great variability, all patients with LBP first sought the advice of GPs. At the time of the survey, six patients were followed by their GP, six by an orthopedist, six by a pain therapist, and six by a physiatrist. Patients with acute cLBP or first exacerbation were followed by GPs or orthopedists, and those with exacerbations of cLBP were referred to pain therapists and physiatrists.

The analysis of interviews showed that patients shared some basic opinions about cLBP. Patients believe that it is not possible to detect a cause of LBP, and therefore to identify a cure. They feel that pain may occur suddenly, and that the disease heavily impacts their HR-QoL. They think that skills and approaches to cLBP, including pharmacological treatments, are very similar across all specialties. Therefore, patients report relying more on the personal relationship than on the scientific background of the physician. Although pharmacologic therapy is not curative in patients’ opinion, it may control pain, avoid flares, and indirectly protect from functionality deterioration. Patients never aim at functionality recovery as a primary objective; they believe that it may occur secondarily to pain improvement.

Although there is a widespread fear of disease flares, patients feel they lack physician’s support for the management of everyday life; they usually conceive and actively implement strategies, also pharmacological treatment with over-the-counter remedies, directed to reduce the impact of pain. This approach usually limits personal activities and social life, with a strong sense of discomfort and inadequacy. This further reduces the HR-QoL, generating a vicious circle.

## 4. Discussion

In this qualitative study, the area of functionality in chronic low back pain (cLBP) as perceived by physicians and patients was investigated. Overall, pain was reported to be a common goal of management, and pharmacological treatment was considered mandatory. Functional impairment was rarely seen as a primary objective of patients’ management and appears to be associated with the use of dedicated scales. Furthermore, patients often come to these specialists when all the others have exhausted their therapeutic capacities, thus finding themselves at the bottom of a supply chain in which not only has pain become chronic, but also highly disabling.

At this point, the problem is cultural, as it is not possible to untie the concept of pain from that of functional recovery. This is true not only in terms of social life and relationships but also of social cost. The worrying fact is that not only some specialists do not consider functional recovery a primary goal, but also patients. These data must be interpreted in the light that there is no correct information on the fearful consequences of pain.

The term “functionality” is used with different meanings by specialists, in relation to the specific aims of their activity. The different specialists involved in the study agreed on the importance of evaluating functionality in clinical practice, but also on the lack of tools able to assess this parameter. In more detail, functionality is described as physical autonomy and mobility by general practitioners (GPs) and orthopedists who usually deal with the early phases of the disease and with exacerbations. Physiatrists and pain therapists interpret functionality as a more comprehensive sphere, corresponding to physical, social, and psychological wellbeing. These specialists are usually in charge of patients with a long history of cLBP, who seek to slow the deterioration of their working and social abilities. They provide interventions that aim at the wellbeing of the patient, and not only at the direct resolution of pain. 

The most recent clinical practice guidelines in Europe have identified a broad spectrum of primarily nonpharmacological treatment alternatives [21]. Nevertheless, when they are called to improve functionality, pharmacological treatment of pain can be used, for example, with the aim of enabling assisted exercise and physiotherapy. 

Opioids must be carefully prescribed and are useful in very selected cases [22,23,24]. Confidence in opioids is lower among orthopedists and GPs, likely due to the fear of adverse events, judged as not frequent but bothersome, and the need for proper patient education. Conversely, confidence in the use of opioids is higher among physiatrists and pain therapists. 

Patients seem to lack more than physicians the perception of the importance of functional recovery and the hope to obtain to a complete health condition. They also remarked on the impact of pain on their HR-QoL, also limiting social and working activities, and the fear of sudden relapses. This suggests that physicians may fail in communicating the hope for a cure, or a durable improvement of the condition. In addition, they do not seem to support enough patients in their efforts to organize their everyday life. Indeed, patients do not rely on their physicians when deciding how to modify their lifestyle.

### Study Limitations

While quantitative research provides a valuable tool for exploring research questions and hypotheses, it has important limitations [25]. Similar to other quantitative research, this study is focused on a specific research question (namely, the functional recovery) and does not provide a comprehensive understanding of the complex chronic low back pain (cLBP) phenomenon. Moreover, some phenomena may be too complex to measure using a brief interview. For example, emotions, attitudes, and beliefs may be difficult to quantify accurately. It involves possible researcher bias as researchers may influence the results of the analysis through their choice of research design, data collection methods, and statistical analysis techniques. Furthermore, quantitative research can identify correlations between variables, but it may be challenging to establish a causal relationship between them. 

The sample size can represent a serious gap. Nevertheless, in qualitative research, what is relevant is how participants are selected. In particular, they must represent their category in terms of beliefs and opinions [26]. In our study, data were analyzed according to macro-groups (e.g., healthcare providers or patients) and smaller subgroups (e.g., physicians who are specialists in a given field). 

Finally, there is an important lack of depth as this quantitative research may not provide a deep understanding of the individual experiences of participants on the investigated cLBP issues. Therefore, we recognize that truly appreciating functionality moves beyond mere words; it requires the inclusion of context to convey its inherent worth. By incorporating patient–specialist pairs, there is potential to uncover shared aspects or disparities in assessing functionality in cases of chronic low back pain. It is important to note that these functional criteria can be subjective when they are tied to the individual patient and evaluated by the specialist based on specific assessment criteria.

## 5. Perspectives

Chronic low back pain (cLBP) is not only a health problem, but a social concern [27]. Notably, its substantial impact on healthcare expenses, work absenteeism, and diminished productivity can severely impact a nation’s economy [28]. Furthermore, a paradox arises from the fact that such a prevalent, significant, and widespread condition such as chronic cLBP is characterized by diverse and occasionally conflicting treatment guidelines, leading to confusion among both patients and pain therapists. [29].

Nevertheless, cLBP can be assumed as a paradigm of chronic pain, a model on which a change can and must begin [30]. Probably, the first change must be cultural, both for patients and physicians [31]. Despite significant efforts to convey the understanding that chronic pain no longer functions solely as a symptom with potential protective effects and acute pain’s physiological significance, further progress is necessary [32].

The next phase involves recognizing that the objective of pain therapy is not solely pain reduction, as measured quantitatively, but also the alleviation of its adverse effects on quality of life [33]. In particular, it implies that the measurement of quality of life in a pain practice must be mandatory [34]. Surprisingly, recent studies indicate that quality of life measurements are often overlooked by early-stage pain professionals, while they are more commonly employed by physiatrists and pain therapists. This holistic approach to patient evaluation should be implemented from the beginning to promptly monitor the outcomes of therapeutic choices [35]. Successfully reaching this goal necessitates a significant investment [36].

On the other hand, investment entails not only training but also the allocation of economic and human resources [37]. Training programs should encompass not only clinical knowledge but also organizational skills and effective communication with other specialists and patients [38]. 

The aim of pain therapy training should be to develop a curriculum that enables accurate diagnoses and appropriate referrals to the most suitable specialists. To achieve this truly ambitious aim, specialists must also be trained from a managerial point of view. However, investing in training alone is insufficient if there is no commitment to establish a well-functioning network between pain therapy centers and general practitioners (GPs), utilizing technological tools such as telemedicine [39,40,41,42]. Technological resources can also help reduce the costs associated with nonpharmacological therapies, which are often inaccessible due to logistical or financial constraints. Relying solely on pharmacological therapy for cLBP is a hasty and ineffective approach [43,44,45]. Furthermore, in the therapeutic process, it is beneficial to involve professionals who specialize in the treatment of low back pain within an integrative medicine framework [46].

Nevertheless, this network will be futile unless accompanied by investment in human resources [19]. Time is the most valuable asset, not just in medicine, and increasing the number of healthcare professionals allows sufficient time to be dedicated to individual patients. This partially explains why quality of life assessments are often neglected during health checkups [47].

Looking ahead, addressing cLBP requires substantial investments in cultural, economic, and human aspects. Nonetheless, considering the potential returns, including economic benefits, these investments seem relatively small. Additionally, the development of standardized and collaborative guidelines is crucial to ensure equal and consistent care for all cLBP patients [48].

## 6. Conclusions

While attention to pain control is now widely recognized, functional recovery is still only a secondary objective for patients with chronic low back pain (cLBP). In order to promote a global approach to cLBP leading the patient from pain control to the improvement of HR-QoL and reestablishment of a healthy condition, some actions could be proposed. These processes should encompass (1) a cultural effort which is necessary to raise attention to the risk of disability for patients with cLBP; (2) a better understanding of the correct use of pharmacological and nonpharmacological strategies among physician; (3) careful pain management for increasing the effectiveness of guided exercises and physiatrist interventions; (4) a strategy aimed at implementing the search of new tools useful in different settings (e.g., telemedicine); (5) definitions and approaches shared among specialties for better management of patients with cLBP, following the right timing.

## Figures and Tables

**Table 1:** Occurrence of different terms on chronic pain or functionality.

Rank *	Item	Occurrences (n)
1	Patient	98
2	Pain	90
3	Functional recovery	29
4	Scale	21
5	Therapy	20
6	Treatment	20
7	Low back pain	19
8	Activity	18
9	Recovery	18
10	Important	18
11	Chronic	18
12	Improve	16
13	Aim	16
14	Cause	16
15	Improvement	15
16	Possible	15
17	Evaluate	15
18	Reduction	15
19	Evaluation	15
20	Psychological	14

Legend: * The first 20 words for occurrence are listed.

**Table 2:** Occurrence of different terms on functionality, according to different specialties.

Rank *	General Practitioner		Orthopedist		Pain Therapist		Physiatrist	
	Item	Occurrences	Item	Occurrences	Item	Occurrences	Item	Occurrences
1	Pain	15	Patient	38	Patient	20	Pain	31
2	Patient	12	Therapy	25	Pain	15	Patient	26
3	Important	10	Pain	19	Social	10	Scale	17
4	Psychological	9	Functional recovery	14	Work	8	Autonomy	9
5	Recovery	7	Low back pain	13	Important	6	Reduction	9
6	Origin	4	Treatment	11	Fundamental	6	Disease	8
7	Mechanical	4	Pharmacological	10	Recover	5	Activity	8
8	Muscular	4	Clinical practice	9	Disability	5	Capability	8
9	Cause	4	Chronic	9	Psychological	5	Improvement	8
10	Psychosocial	3	Objective	9	Working activity	5	Chronic pain	8

Legend. * The first 10 words for occurrence are listed.

## Data Availability

The datasets used and/or analyzed during the current study are available from Arturo Cuomo on reasonable request.

## References

[1] Buchbinder R., van Tulder M., Öberg B., Costa L.M., Woolf A., Schoene M., Croft P. (2018). Lancet Low Back Pain Series Working Group Low Back Pain: A Call for Action. Lancet.

[2] Hartvigsen J., Hancock M.J., Kongsted A., Louw Q., Ferreira M.L., Genevay S., Hoy D., Karppinen J., Pransky G., Sieper J. (2018). What Low Back Pain Is and Why We Need to Pay Attention. Lancet.

[3] Gibbs D., McGahan B.G., Ropper A.E., Xu D.S. (2023). Back Pain: Differential Diagnosis and Management. Neurol. Clin..

[4] Wilson R., Pryymachenko Y., Abbott J.H., Dean S., Stanley J., Garrett S., Mathieson F., Dowell A., Darlow B. (2023). A Guideline-Implementation Intervention to Improve the Management of Low Back Pain in Primary Care: A Difference-in-Difference-in-Differences Analysis. Appl. Health Econ. Health Policy.

[5] Enomoto H., Sasaki N., Fujikoshi S., Yoshikawa A., Tsuji T., Takeshita K. (2019). Relationship between Pain Alleviation and Disease-Specific Health-Related Quality of Life Measures in Patients with Chronic Low Back Pain Receiving Duloxetine: Exploratory Post Hoc Analysis of a Japanese Phase 3 Randomized Study. J. Am. Acad. Orthop. Surg. Glob. Res. Rev..

[6] Kabała T., Giemza C. (2020). Efficiency of Active Therapy for Low Back Pain in Elderly Men. J. Back Musculoskelet. Rehabil..

[7] Tamer S., Öz M., Ülger Ö. (2017). The Effect of Visceral Osteopathic Manual Therapy Applications on Pain, Quality of Life and Function in Patients with Chronic Nonspecific Low Back Pain. J. Back Musculoskelet. Rehabil..

[8] Chou R., Deyo R., Friedly J., Skelly A., Weimer M., Fu R., Dana T., Kraegel P., Griffin J., Grusing S. (2017). Systemic Pharmacologic Therapies for Low Back Pain: A Systematic Review for an American College of Physicians Clinical Practice Guideline. Ann. Intern. Med..

[9] Visconti C., Mastroluca A., Varano L., Del Gaudio A. (2019). Tapentadol Prolonged Release in Association with Analgesic Radiofrequency for the Treatment of Chronic Lumbar Radicular Pain: An Observational, Prospective Study. Eur. Rev. Med. Pharmacol. Sci..

[10] Orfei M., Milia P., Caserio M., Bagaphou T.C. (2019). Efficacy and Tolerability of Tapentadol Prolonged Release during Rehabilitation: A Prospective, Observational Study. Eur. Rev. Med. Pharmacol. Sci..

[11] Coluzzi F., Ruggeri M. (2014). Clinical and Economic Evaluation of Tapentadol Extended Release and Oxycodone/Naloxone Extended Release in Comparison with Controlled Release Oxycodone in Musculoskeletal Pain. Curr. Med. Res. Opin..

[12] Treede R.-D., Rief W., Barke A., Aziz Q., Bennett M.I., Benoliel R., Cohen M., Evers S., Finnerup N.B., First M.B. (2019). Chronic Pain as a Symptom or a Disease: The IASP Classification of Chronic Pain for the International Classification of Diseases (ICD-11). Pain.

[13] Karra R., Holten-Rossing S., Mohammed D., Parmeggiani L., Heine M., Namnún O.C. (2021). Unmet Needs in the Management of Functional Impairment in Patients with Chronic Pain: A Multinational Survey. Pain Manag..

[14] Cascella M., Miceli L., Cutugno F., Di Lorenzo G., Morabito A., Oriente A., Massazza G., Magni A., Marinangeli F., Cuomo A. (2021). A Delphi Consensus Approach for the Management of Chronic Pain during and after the COVID-19 Era. Int. J. Environ. Res. Public Health.

[15] Pyo J., Lee W., Choi E.Y., Jang S.G., Ock M. (2023). Qualitative Research in Healthcare: Necessity and Characteristics. J. Prev. Med. Public Health.

[16] Crombez P., Bron D., Michiels S. (2019). Multicultural Approaches of Cancer Pain. Curr. Opin. Oncol..

[17] Nichols V.P., Ellard D.R., Griffiths F.E., Kamal A., Underwood M., Taylor S.J.C. (2017). CHESS team The Lived Experience of Chronic Headache: A Systematic Review and Synthesis of the Qualitative Literature. BMJ Open.

[18] Vollert J., Kleykamp B.A., Farrar J.T., Gilron I., Hohenschurz-Schmidt D., Kerns R.D., Mackey S., Markman J.D., McDermott M.P., Rice A.S.C. (2023). Real-World Data and Evidence in Pain Research: A Qualitative Systematic Review of Methods in Current Practice. Pain Rep..

[19] Vittori A., Petrucci E., Cascella M., Innamorato M., Cuomo A., Giarratano A., Petrini F., Marinangeli F. (2021). Pursuing the Recovery of Severe Chronic Musculoskeletal Pain in Italy: Clinical and Organizational Perspectives from a SIAARTI Survey. J. Pain Res..

[20] Cascella M., Vittori A., Petrucci E., Marinangeli F., Giarratano A., Cacciagrano C., Tizi E.S., Miceli L., Natoli S., Cuomo A. (2022). Strengths and Weaknesses of Cancer Pain Management in Italy: Findings from a Nationwide SIAARTI Survey. Healthcare.

[21] Corp N., Mansell G., Stynes S., Wynne-Jones G., Morsø L., Hill J.C., van der Windt D.A. (2021). Evidence-Based Treatment Recommendations for Neck and Low Back Pain across Europe: A Systematic Review of Guidelines. Eur. J. Pain.

[22] Knezevic N.N., Candido K.D., Vlaeyen J.W.S., Van Zundert J., Cohen S.P. (2021). Low Back Pain. Lancet.

[23] Biancuzzi H., Dal Mas F., Brescia V., Campostrini S., Cascella M., Cuomo A., Cobianchi L., Dorken-Gallastegi A., Gebran A., Kaafarani H.M. (2022). Opioid Misuse: A Review of the Main Issues, Challenges, and Strategies. Int. J. Environ. Res. Public Health.

[24] Cascella M., Monaco F., Nocerino D., Chinè E., Carpenedo R., Picerno P., Migliaccio L., Armignacco A., Franceschini G., Coluccia S. (2022). Bibliometric Network Analysis on Rapid-Onset Opioids for Breakthrough Cancer Pain Treatment. J. Pain Symptom Manag..

[25] Moser A., Korstjens I. (2018). Series: Practical Guidance to Qualitative Research. Part 3: Sampling, Data Collection and Analysis. Eur. J. Gen. Pract..

[26] Colorafi K.J., Evans B. (2016). Qualitative Descriptive Methods in Health Science Research. Health Environ. Res. Des. J..

[27] Natoli S., Vittori A., Cascella M., Innamorato M., Finco G., Giarratano A., Marinangeli F., Cuomo A. (2022). Raising Awareness on the Clinical and Social Relevance of Adequate Chronic Pain Care. Int. J. Environ. Res. Public Health.

[28] Miki T., Kondo Y., Kurakata H., Takebayashi T., Samukawa M. (2023). Physical Therapist-Led Interventions Based on the Biopsychosocial Model Provide Improvement in Disability and Pain for Spinal Disorders: A Systematic Review and Meta-Analysis. PM&R.

[29] Nicol V., Verdaguer C., Daste C., Bisseriex H., Lapeyre É., Lefèvre-Colau M.-M., Rannou F., Rören A., Facione J., Nguyen C. (2023). Chronic Low Back Pain: A Narrative Review of Recent International Guidelines for Diagnosis and Conservative Treatment. J. Clin. Med..

[30] Urits I., Burshtein A., Sharma M., Testa L., Gold P.A., Orhurhu V., Viswanath O., Jones M.R., Sidransky M.A., Spektor B. (2019). Low Back Pain, a Comprehensive Review: Pathophysiology, Diagnosis, and Treatment. Curr. Pain Headache Rep..

[31] Mescouto K., Olson R.E., Hodges P.W., Setchell J. (2022). A Critical Review of the Biopsychosocial Model of Low Back Pain Care: Time for a New Approach?. Disabil. Rehabil..

[32] Wade D.T., Halligan P.W. (2017). The Biopsychosocial Model of Illness: A Model Whose Time Has Come. Clin. Rehabil..

[33] Mroczek B., Łubkowska W., Jarno W., Jaraczewska E., Mierzecki A. (2020). Occurrence and Impact of Back Pain on the Quality of Life of Healthcare Workers. Ann. Agric. Environ. Med..

[34] Wettstein M., Eich W., Bieber C., Tesarz J. (2019). Pain Intensity, Disability, and Quality of Life in Patients with Chronic Low Back Pain: Does Age Matter?. Pain Med..

[35] Cuomo A., Cascella M., Forte C.A., Bimonte S., Esposito G., De Santis S., Cavanna L., Fusco F., Dauri M., Natoli S. (2020). Careful Breakthrough Cancer Pain Treatment through Rapid-Onset Transmucosal Fentanyl Improves the Quality of Life in Cancer Patients: Results from the BEST Multicenter Study. J. Clin. Med..

[36] Lorio M.P., Beall D.P., Calodney A.K., Lewandrowski K.-U., Block J.E., Mekhail N. (2023). Defining the Patient with Lumbar Discogenic Pain: Real-World Implications for Diagnosis and Effective Clinical Management. J. Pers. Med..

[37] Vittori A., Cascella M., Petrucci E., Cortegiani A., Bignami E.G., Innamorato M.A., Cuomo A., Torrano V., Petrini F., Giarratano A. (2023). Strategies to Build and Maintain Competence in Pain Management: Insights from a SIAARTI Survey on Educational Needs among Italian Anesthesiologists. Pain Pract..

[38] Kongsted A., Ris I., Kjaer P., Hartvigsen J. (2021). Self-Management at the Core of Back Pain Care: 10 Key Points for Clinicians. Braz. J. Phys. Ther..

[39] Moreno-Ligero M., Moral-Munoz J.A., Salazar A., Failde I. (2023). MHealth Intervention for Improving Pain, Quality of Life, and Functional Disability in Patients with Chronic Pain: Systematic Review. JMIR mHealth uHealth.

[40] Miceli L., Dal Mas F., Biancuzzi H., Bednarova R., Rizzardo A., Cobianchi L., Holmboe E.S. (2022). Doctor@Home: Through a Telemedicine Co-Production and Co-Learning Journey. J. Cancer Educ..

[41] Bhaskar S., Bradley S., Chattu V.K., Adisesh A., Nurtazina A., Kyrykbayeva S., Sakhamuri S., Yaya S., Sunil T., Thomas P. (2020). Telemedicine across the Globe-Position Paper From the COVID-19 Pandemic Health System Resilience PROGRAM (REPROGRAM) International Consortium (Part 1). Front. Public Health.

[42] Kvedar J., Coye M.J., Everett W. (2014). Connected Health: A Review of Technologies and Strategies to Improve Patient Care with Telemedicine and Telehealth. Health Aff..

[43] Cascella M., Marinangeli F., Vittori A., Scala C., Piccinini M., Braga A., Miceli L., Vellucci R. (2021). Open Issues and Practical Suggestions for Telemedicine in Chronic Pain. Int. J. Environ. Res. Public Health.

[44] Cascella M., Schiavo D., Grizzuti M., Romano M.C., Coluccia S., Bimonte S., Cuomo A. (2023). Implementation of a Hybrid Care Model for Telemedicine-Based Cancer Pain Management at the Cancer Center of Naples, Italy: A Cohort Study. In Vivo.

[45] Cascella M., Coluccia S., Monaco F., Schiavo D., Nocerino D., Grizzuti M., Romano M.C., Cuomo A. (2022). Different Machine Learning Approaches for Implementing Telehealth-Based Cancer Pain Management Strategies. J. Clin. Med..

[46] Maiers M., Salsbury S.A. (2022). “Like Peanut Butter and Jelly”: A Qualitative Study of Chiropractic Care and Home Exercise among Older Adults with Spinal Disability. Arthritis Care Res..

[47] Toma A.-O., Boeriu E., Decean L., Bloanca V., Bratosin F., Levai M.C., Vasamsetti N.G., Alambaram S., Oprisoni A.L., Miutescu B. (2023). The Effects of Lack of Awareness in Age-Related Quality of Life, Coping with Stress, and Depression among Patients with Malignant Melanoma. Curr. Oncol..

[48] Occhigrossi F., Carpenedo R., Leoni M.L.G., Varrassi G., Chinè E., Cascella M., Compain research group (2023). Delphi-Based Expert Consensus Statements for the Management of Percutaneous Radiofrequency Neurotomy in the Treatment of Lumbar Facet Joint Syndrome. Pain Ther..

